# Lifestyle changes of Japanese people on overseas assignment in Michigan, USA

**DOI:** 10.1186/1447-056X-8-7

**Published:** 2009-07-16

**Authors:** Kazuya Kitamura, Michael D Fetters, Kiyoshi Sano, Juichi Sato, Nobutaro Ban

**Affiliations:** 1 Kachigawa Family Clinic, 1-3 Matsushin-cho, Kasugai-shi, Aichi 486-0931, Japan; 2 Department of Family Medicine, University of Michigan, Ann Arbor, USA; 3 Family Practise, American Hospital of Paris, Paris, France; 4 Department of General Medicine, Nagoya University Hospital, Nagoya, Japan

## Abstract

**Background:**

Temporary work assignments in the United States (US) are widely considered to have negative health outcomes on Asians mostly due to adverse changes in diet and exercise, though there is little research on this phenomenon. This study investigated the impact of lifestyle changes on the biological and psychological health and health behaviours of Japanese people on temporary assignments in the US.

**Methods:**

In this cross sectional survey, we distributed a 38 item self-administered questionnaire addressing health habits, mental health function, lifestyle changes and dietary habits to adult Japanese patients presenting for general physicals at a family medicine clinic serving Japanese patients. We conducted simple statistics and regression analysis between length of stay and other health outcomes to determine whether length of residence in the US was predictive of negative lifestyle changes.

**Results:**

Most participants reported increased caloric intake, weight gain, and less exercise. They also reported increased time with family. More women than men reported physical symptoms and anxiety related to stress. Smoking and alcohol intake were essentially unchanged. No associations were identified between length of residence in the US and health lifestyle habits or other health outcomes.

**Conclusion:**

Negative lifestyle changes occur in diet and exercise for overseas Japanese people, but a positive change in increased family time was found. Women appear to be at a greater risk for somatic disorders than men. As duration of stay does not appear predictive of adverse changes, clinicians should advise patients going abroad of these risks regardless of the term of the work assignment.

## Introduction

An individual's health behaviours including eating habits, exercise, rest, smoking, and alcohol consumption affect the development of many diseases [1-6]. Due to social, cultural and environmental differences, Asians who live overseas for temporary work assignments will likely experience sudden lifestyle changes. Based on clinical observations, these sudden lifestyle changes are suspected to be related to adverse health outcomes. Due to the globalization of business, an increasing number of Japanese people are transferred to other countries where there may be enormous environmental pressures to change their health behaviours and adjust to a different culture[7] It has been estimated that there are about 300,000 Japanese people living in the United States (US), and two-thirds of them are on temporary assignment for business[8]

Health professionals caring for Japanese people need information about the influence of lifestyle changes in order to guide their counselling efforts to promote healthy behaviours. While previous authors have addressed the influence of temporary assignment on lifestyle changes, these reports have been printed in Japanese and most are not published as original research [9-14] Previous sentinel research such as the Ni-Hon-San study attributes differences in coronary heart disease and cerebrovascular disease among Japanese people living in Japan and Hawaii to differences in dietary protein and animal fat [15]. Subsequent epidemiological studies have further explored lifestyle factors as contributors to differences in coronary artery calcification in Japanese men in Hawaii and Japan [16] and stroke in Finland and Japan [17] though such studies have primarily focused on cardiovascular disease (CVD).

Given this relatively narrow focus on CVD in previous research, we sought to investigate the influence of lifestyle changes on both the biological and psychological health affecting Japanese people on temporary overseas assignment in the US. We hypothesized there would be adverse dietary changes (higher caloric intake) and reduced physical activity, but decreased alcohol intake and tobacco use due to fewer occasions to drink alcohol for business and an anti-smoking work environment, respectively.

## Materials and methods

In this cross-sectional survey, participants answered structured questions about their lifestyle changes and health behaviours after coming to the US. The investigation was conducted through the University of Michigan Japanese Family Health Program (JFHP) [18]. This family medicine program serves the needs of Japanese families who are in the US on temporary assignment. Subjects who had been in the US for one or more years and presented for a health maintenance examination (HME) from the JFHP during the study period were eligible to participate. We offered participation to all Japanese employees and their adult family members who met enrolment criteria from May 16 to August 31, 2001.

One week prior to their scheduled HME, we sent participants by mail an invitation to participate and a survey instrument (both written in Japanese). Interested subjects were advised to complete the survey instruments prior to the visit. Comprehensive, project specific, written informed consent was obtained at the time of the HME visit. The sample included individuals who were already registered as patients, and individuals who newly registered as patients, though the purpose of the visit when the survey was conducted was to have a HME.

We asked each participant to complete a questionnaire with items addressing demographics, personal health habits including diet [19], physical activity [20], tobacco use, alcohol consumption (assessed with CAGE [21] and Kurihama Alcoholism Screening Test (KAST) [22]-an instrument developed and standardized in Japan), mental health status (using the GHQ28-General Health Questionnaire, standardized Japanese version) [23], their lifestyle changes after coming to the US and any changes in chronic medical problems. To assess dietary habits, we asked participants to record their dietary intake one weekday and one weekend day. We distributed a diet record manual in which common Japanese foods were listed by popular names, and with examples of standard quantities for each food listed.

Data were analyzed using SPSS (Statistical Package for Social Sciences). In our analysis, we analyzed data from men and women separately because men are immersed in US society through their workplace, whereas women, usually housewives, are more likely to be socially isolated because of language and transportation issues. To determine whether length of residence in the US was an important factor, we conducted regression analysis between length of stay and other health outcomes.

The Institutional Review Board of the University of Michigan Health System approved this project.

## Results

We distributed the questionnaire to 128 patients. Nine individuals cancelled their appointments and ten were excluded because they had been in the US for less than 12 months, and one declined participation. As a result, 108 (response rate 84.4%) patients were included in the analysis. The demographics of the participants are listed in Table 1. All male participants were company employees who were accompanied by family members.

**Table 1:** Participant demographics of overseas Japanese men and women in Michigan, USA

N = 108	Male	Female
Number	63	45
Age		
range	27–61	27–60
average ± SD	40.1	37.2
Height (average ± SD) cm	169.9 ± 4.9	158.0 ± 4.9
Weight (average μ ± SD) kg	69.6 ± 7.1	52.8 ± 7.4
BMI (average ± SD) kg/m^2^	24.2 ± 2.5	21.2 ± 3.2
Employee	63	2
Marital status		
single	1	1
married	62	44
have children	55	37

### Diet & Exercise

The daily energy intake and exercise of participants are depicted in Table 2. The daily energy intake from fat is greater than one third of total energy intake among men and women on weekdays and weekends. Some participants commented they had fewer opportunities to eat fish in the US than in Japan. A few participants among men and women expended more than 10% of their energy of daily activities by exercising. Moreover, only 20% expended more than 10% of their total daily energy expenditure through the combined activities of exercising and commuting. Many participants reported walking less in the US because they drive from their garage to the parking lot of their company.

**Table 2:** Total energy intake and daily exercise of overseas Japanese men and women in Michigan, USA

	Male (N = 63)	Female (N = 45)	Total (N = 108)	p-value
Total energy intake and percentage from fat				
weekdays energy (kcal)	1813	1796	1806	0.41
% fat	36.5	34.8	35.8	0.40
weekends energy (kcal)	1851	1684	1781	0.27
% fat	35.5	36.6	35.3	0.76
				
Daily exercise as percentage of total daily physical activity	n (%)	n (%)	n (%)	
0%	10 (16)	12 (27)	22 (20)	0.16
1 to <10%	50 (79)	30 (67)	80 (74)	0.14
10% and more	3 (5)	3 (7)	6 (6)	0.69

### Smoking and Alcohol

The smoking and drinking habits of participants are presented in Table 3. About one third of the male participants formerly smoked and another third were current smokers. Most female participants reported having never smoked. Nine in 10 males and 1 in 2 females reported drinking alcohol. About 1 in 10 men had positive screen for alcoholism using the KAST standard while 1 in 5 men were positive by CAGE questionnaire using a score of 2.

**Table 3:** Exercise, smoking and alcoholism of overseas Japanese men and women in Michigan, USA

	Male (N = 63)	Female (N = 45)	Total (N = 108)	p-value
	n (%)	n (%)	n (%)	
Smoking status				
current smoker	20 (32)	3 (7)	23 (21)	<0.01
former smoker	22 (35)	1 (2)	23 (21)	<0.01
never smoked	21 (33)	41 (91)	62 (57)	<0.01
Drinking status	57 (90)	25 (56)	82 (76)	<0.01
Positive alcoholism screen*				
CAGE	14 (22)	0 (0)	14 (13)	<0.01
KAST	6 (10)	0 (0)	6 (6)	0.04

* positive screen defined by CAGE≧2; KAST≧0

### Mental Health

With regard to mental health status (Table 4), about one-third of female participants reported more than moderate somatic symptoms, and scored positively for anxiety and insomnia. When we compared mental health scores for men and women, women were more likely to have physical symptoms related to stress than men.

**Table 4:** Mental heath assessment based on GHQ scores of overseas Japanese men and women in Michigan, USA

Positive mental illness screen*	Male (N = 63)	Female (N = 45)	Total (N = 108)	p-value
	n (%)	n (%)	n (%)	
Somatic symptoms	6 (10)	13 (29)	19 (18)	<0.01
Anxiety/insomnia	9 (14)	12 (27)	21 (20)	0.11
Social distress	6 (10)	6 (13)	12 (11)	0.54
Depression	1 (2)	2 (4)	3 (3)	0.57

*mental illness screen based on GHQ

### Self-reports of Lifestyle Changes

Participants' self-reports of lifestyle changes are depicted in Table 5. More than half of participants reported increased energy intake, body weight, and time that they spent with their family. On the other hand, most stated snacking between meals, alcohol consumption, tobacco use, working hours, overtime and office visits to a doctor were almost the same as when they lived in Japan.

**Table 5:** Self-reported lifestyle changes in Michigan, USA

Compared to life in Japan: N = 108	Participants responding n	Much/somewhat more n (%)	Almost the same n (%)	Somewhat/much less n (%)
Calorie intake	107	71 (65)	30 (28)	6 (6)
Hours with family	104	65 (63)	29 (28)	10 (10)
Body weight	107	60 (56)	32 (30)	15 (14)
Overtime work	64	21 (33)	23 (36)	20 (31)
Snacking between meals	103	34 (33)	67 (65)	2 (2)
Physical exercise	108	30 (30)	32 (30)	46 (43)
Working hours (includes housework)	105	30 (29)	44 (42)	31 (30)
Alcohol consumption	81	23 (28)	45 (56)	13 (13)
Number of vacation days	81	23 (28)	32 (40)	26 (32)
Number of sick/family days	67	14 (21)	27 (40)	26 (39)
Number of cigarettes	22	3 (14)	10 (48)	9 (43)
Number of office visits to a doctor	103	5 (5)	64 (62)	34 (33)
Chronic medical problem(s)	24	3 (13)	14 (58)	7 (29)

### Lengths of stay and health outcomes

We examined for an association between length of residence in the US and health lifestyle habits and other health outcomes. In our regression analysis, we found no statistical differences.

## Discussion

Japanese people on temporary assignment in the US reported lifestyle changes that influenced their health behaviours and health indices. Adverse changes included increased energy intake, body weight, and decreased physical activity as hypothesized. Psychiatric illness was low, though higher among women than men. Most chronic medical problems were unchanged. To our surprise, overall smoking and drinking levels did not appear to change, but the pattern of drinking changed.

The physical activity and dietary data revealed less physical activity and more energy intake from fat. Miyazaki et al[13] reported that the Body Mass Index (BMI) of many Japanese people on temporary assignment overseas (3–5 years) tended to decrease regardless of country. In our study however, these Japanese people in the US reported increasing body weight. While most participants reported eating Japanese food almost every day, men in this sample might be affected more by lifestyle changes in the US such as increased fat consumption and less walking due to their greater direct participation in US society through the workplace. Although the mean BMI in the participants increased by self-report, it did remain within a normal range. Still the duration of stay was relatively short (the average was 33 months) and it is plausible it could reach an abnormal range after longer duration, though this is speculative.

Women had more physical symptoms related to stress and insomnia than men. The low rate of depression found is somewhat surprising given previous research demonstrating similar rates of depression in Japan and other countries on standardized screening instruments in primary clinics [24,25], and from Japanese clinics revealing high rates of somatic symptoms, and physical symptoms associated with depression in about 13–15% of patients [26-28]. This discrepancy in symptom versus depression reports is similar to research conducted in international clinics where patients with depression who had only physical symptoms ranged from 45–95% with 11% denying depression despite direct questioning [29]. The trend for higher psychological symptoms among women than men in the sample is also consistent with US Department of Health and Human Services reports of women being nearly twice as likely as men to experience depression [30]. Most women participants accompanied their husbands to the US and many had children. Wives likely face different difficulties related to language, culture and raising children than their husbands encounter in the workplace. These data emphasize the importance of developing mental health support systems, especially for women.

We did not find an association with length of time lived in the US and health lifestyle habits and health outcomes, even though many participants reported many behavioural changes after moving from Japan. The lack of association between duration of living in the US and lifestyle changes and health outcomes may be attributed to the relatively narrow variation in the number of years in the US, or the event of just moving to US might affect the lifestyle changes of all similarly.

A potential limitation of this research is selection bias. The study was conducted exclusively in Southeastern Michigan. Japanese people on work assignment here are usually affiliated with the automotive industry and may not be representative of overseas employees who are assigned in other US regions. Second, the self-reported changes may be influenced by recall bias and it is difficult to assess how this might differ according to length of stay. Participants who have been in the US less time might be more aware of lifestyle changes and their influence than individuals for who more time has elapsed. However, the latter group may have more stability in their changes. Third, we did not include children as participants.

A prospective cohort study with baseline biomarker assessments prior to departure, periodically after arrival abroad, and after return to Japan could more definitively document the impact of overseas assignment on the lifestyle changes of Japanese people and permit calculation of absolute risk as a consequence of all lifestyle changes. A larger sample size is needed to examine more fully less prevalent problems such as smoking behaviours and mental illness. Clinical data on BMI, laboratory values, caloric data analysis correlated with self-reports could lead to a better understanding of the occurrence and patterns of health behaviour changes. The impact of lifestyle changes on children, particularly given the epidemic of childhood obesity in the US, should be investigated in future research.

After coming to the US, these Japanese people experienced many lifestyle changes. Adverse changes included increased energy intake, body weight, and decreased physical activity as we expected. A beneficial change was the increased time with family. Some reported increased time with their family on holidays because they worked fewer holidays compared to Japan. None reported worsening of chronic medical problems.

These data confirm widespread perceptions among Asians that there are adverse health risks due to lifestyle changes during temporary work assignments in the United States. Clinicians who provide care for patients who go abroad for assignments in the US have an opportunity to counsel about potential adverse health changes and can promote healthy behaviours. Increased caloric intake, weight gain and physical inactivity appear to be the greatest risks. Surprisingly, even a short duration carries this risk. Future research should examine the generalizability of these findings to other populations. Prospective studies that include biomarkers are needed to conclusively illustrate if duration of assignment is associated with worse outcomes.

## Summary of implications for GPs

In the global economy, an increasing number of Asians travel abroad for work, yet little research has addressed what lifestyle changes will affect them. Among Japanese people on temporary assignment in the US, most reported negative lifestyle changes such as increased caloric intake, weight gain, and less exercise. A positive outcome of living in the US was an increase in time to spend with the family. More women than men reported physical symptoms and anxiety related to stress. Smoking and alcohol intake were essentially unchanged. Duration of assignment abroad was not predictive of these changes. While this sample was limited to Japanese patients, other Asians who travel to the US may be at risk for similar lifestyle changes. General practitioners can promote the health of their patients who go abroad by counselling them about potential health risks of lifestyle changes.

## Competing interests

The authors declare that they have no competing interests.

## Authors' contributions

KK and JS contributed to the conception and study design, performed data analysis, interpretation. KS contributed to the conception and study design, and data collection. MDF contributed to the conception and study design, data collection, and critical editing of the manuscript. NB participated in the study design and critical revision of the manuscript.

All authors read and approved the final manuscript.
